# Rationale and Challenges for a New Instrument for Remote Measurement of Negative Symptoms

**DOI:** 10.1093/schizbullopen/sgae027

**Published:** 2024-10-18

**Authors:** David Gordon Daniel, Alex S Cohen, Philip D Harvey, Dawn I Velligan, William Z Potter, William P Horan, Raeanne C Moore, Stephen R Marder

**Affiliations:** Signant Health, Blue Bell, PA, USA; Bioniche Global Development, LLC, McLean, VA, USA; George Washington University, Washington, DC, USA; Louisiana State University, Baton Rouge, LA, USA; University of Miami, Miami, FL, USA; University of Texas Health Science Center at San Antonio, San Antonio, TX, USA; Independent Expert, Philadelphia, PA, USA; Karuna—A Bristol Myers Squibb Company, Boston, MA, USA; San Diego State University, San Diego, CA, USA; Semel Institute for Neuroscience at UCLA and the VA Desert Pacific Mental Illness Research, Education and Clinical Center, Los Angeles, CA, USA

**Keywords:** schizophrenia, negative symptoms, remote measurement, rating scale, digital measures, ecological momentary assessment

## Abstract

There is a broad consensus that the commonly used clinician-administered rating scales for assessment of negative symptoms share significant limitations, including (1) reliance upon accurate self-report and recall from the patient and caregiver; (2) potential for sampling bias and thus being unrepresentative of daily-life experiences; (3) subjectivity of the symptom scoring process and limited sensitivity to change. These limitations led a work group from the International Society of CNS Clinical Trials and Methodology (ISCTM) to initiate the development of a multimodal negative symptom instrument. Experts from academia and industry reviewed the current methods of assessing the domains of negative symptoms including diminished (1) affect; (2) sociality; (3) verbal communication; (4) goal-directed behavior; and (5) Hedonic drives. For each domain, they documented the limitations of the current methods and recommended new approaches that could potentially be included in a multimodal instrument. The recommended methods for assessing negative symptoms included ecological momentary assessment (EMA), in which the patient self-reports their condition upon receipt of periodic prompts from a smartphone or other device during their daily routine; and direct inference of negative symptoms through detection and analysis of the patient’s voice, appearance or activity from audio/visual or sensor-based (eg, global positioning systems, actigraphy) recordings captured by the patient’s smartphone or other device. The process for developing an instrument could resemble the NIMH MATRICS process that was used to develop a battery for measuring cognition in schizophrenia. Although the EMA and other digital measures for negative symptoms are at relatively early stages of development/maturity and development of such an instrument faces substantial challenges, none of them are insurmountable.

## Introduction

Negative symptoms are strongly related to functional outcomes and quality of life in patients with schizophrenia, and their treatment represents an important unmet need.^1^ In phase 3 clinical trials with schizophrenia patients with predominantly negative symptoms, attainment of robust placebo-drug separation has been challenging, and no pharmacological treatments have, to date, clearly demonstrated effectiveness.^1^ Factor analyses suggest that negative symptoms can be broadly categorized as diminished expression and avolition/apathy and further subdivided into 5 domains representing reductions in affect, sociality, verbal communication, goal-directed behavior, and hedonic drives.^1,2^ Among the challenges for the development of new therapies is the possibility that these domains are underpinned, at least in part, by alterations in different neural circuitry and neurotransmitters and that bespoke pharmacological interventions may be required to treat them optimally. Currently, available measurement tools for the assessment of these domains have significant limitations or are in early investigational stages.^3^

The most used clinician-administered rating scales for assessment of negative symptoms are the Marder negative factor derived from the Positive and Negative Syndrome Scale (PANSS), the Negative Symptom Assessment-16 (NSA-16), the Brief Negative Symptom Scale (BNSS), and the Clinical Assessment Interview for Negative Symptoms (CAINS).^4–11^ All have favorable psychometric qualities, however, as shown in table 1, they differ markedly in their approaches to measuring the 5 currently recognized domains of negative symptoms. Moreover, all share significant limitations, including (1) reliance upon accurate self-report and recall from the patient and caregiver; (2) potential for sampling bias in that patients’ responses may be influenced by the office environment and their relationship with the interviewer and thus may not be representative of behavior in daily life; and (3) subjectivity of the symptom scoring process which involves weighing and integrating potentially contradictory reports from the patient and informant and direct observation.

**Table 1.** Construct and Item-Level Comparison

Domain	PANSS Negative	PANSS Marder	NSA-16	BNSS	CAINS	Method
Diminished affect	Blunted affect	Blunted affect	Affect: reduced modulation of intensity	Facial expression Vocal expression Expressive gestures	Facial expression Vocal expression Expressive gestures	Observational
			Emotion: reduced range			Reported
			Affect: reduced display on demand			Observational
Diminished sociality	Emotional withdrawal	Emotional withdrawal				Observational
	Poor rapport	Poor rapport	Poor rapport with interviewer			Observational
	Passive/apathetic social withdrawal	Passive/apathetic social withdrawal	Reduced social drive	Asociality behavior		Reported
				Asociality internal experience		Reported
					Family relationships	
					Friendships	
					Frequency of past pleasurable social activities	
					Frequency of expected pleasure from social activities	
		Active social avoidance				
			Sexual interest			
Diminished verbal communication	Lack of spontaneity and flow of conversation	Lack of spontaneity and flow of conversation		Spontaneous elaboration		Observational
			Prolonged time to respond			Observational
			Restricted speech quantity	Quantity of speech	Quantity of speech	Observational
			Impoverished speech content			Observational
			Inarticulate speech			Observational
				Vocal expression	Vocal expression	Observational
Diminished goal-directed behavior and interest			Reduced sense of purpose			Reported
			Reduced interest			Reported
			Reduced daily activity	Avolition behavior	Hobbies recreation pastimes	Reported
			Poor grooming and hygiene			Observational
Diminished hedonic drives				Frequency of pleasure during activities	Frequency of pleasure from recreational activities	
				Intensity of pleasure during activities		
					Motivation for work school activities	
					Frequency of expected pleasure from vocational activities	
				Intensity of expected pleasure from future activities	Frequency of expected pleasure from recreational activities	
				Avolition internal experience		
		Motor retardation	Slowed movements			Observational
				Distress		Reported
	Difficulty in abstract thinking					Observational
	Stereotyped thinking					

In recognition of the issues with the current clinician-administered rating scales, the Negative Symptoms Work Group of the International Society of CNS Clinical Trials and Methodology (ISCTM) convened in February 2023 and reached a consensus that the time was right for the development of a new multimodal instrument for evaluation of negative symptoms. There was agreement among the attendees that remote monitoring might have the potential to overcome many of the limitations of the clinician-administered rating scales in the evaluation of negative symptoms. It was noted that, to date, no modalities for remote assessment of negative symptoms have been validated to the extent that they would be acceptable as primary outcome measures in registration clinical trials. The current primary investigational modalities for remote assessment of negative symptoms are ecological momentary assessment (EMA), in which the patient self-reports in multiple domains upon receipt of periodic prompts from a smartphone or other device during their daily routine; and direct inference of negative symptoms through detection and analysis of the patient’s voice, appearance, or activity by way of the patient’s smartphone or other device. The committee expressed optimism that compared to clinician-administered rating scales, frequent assessment using these modalities in the patient’s own environment could have the potential to reduce the risk of recall error, reduce bias from face-to-face interaction with the investigator and the office environment, reduce reliance on the subjective judgment of the investigator, and potentially identify new treatment targets.

Subsequently, volunteers from the committee, including representatives of academia and the pharmaceutical industry, met by teleconference over the course of 2023 to (1) critique how each of the recognized domains of negative symptoms (table 1) are currently measured; (2) discuss the limitations of the current methods in measuring each domain; (3) suggest newer approaches to measuring each domain; and (4) make recommendations for measuring each domain in a new multimodal instrument. Those findings were presented in summary form to the Negative Symptoms Work Group of the International Society of CNS Clinical Trials and Methodology (ISCTM) convened in February 2024. The results of those proceedings are described below.

## Diminished Affect

### Current Assessment Methods

Affect involves the use of nonverbal behavioral expression to share information between people, such as through facial expression, vocal modulation, head and hand movement, and other body-expressive gestures.^12^ In clinical practice, blunted affect is primarily detected through 3 methods: interactions with a trained professional during an unstructured mental status exam, structured clinical interviews and rating scales, and information collected with performance-based (eg, Social Skills Performance Assessment) tests. The latter 2 approaches are generally considered to offer face validity and acceptable psychometrics for differentiating gross abnormalities in affect. Patients with negative symptoms are generally rated as having deficits in affective expression on the order of 2–4 SDs compared to patients without negative symptoms and nonpsychiatric controls.^13^ These ratings tend to be stable over extended observation periods and are correlated with various measures of functioning, treatment response, and prognosis.^14,15^ Additionally, several self-report measures of emotional expression exist; and many self-report measures of clinical high-risk psychosis and schizotypy include measures of “constricted affect.”^16–18^

### Limitations to Current Methods

As with other current measures of negative symptoms, affective assessment requires significant resources from both patients and researchers, often has questionable ecological validity generalizing to behavior beyond the clinical interview, and is prone to biases both within and between raters. An additional challenge involves the reality that affective expression is highly dynamic both within people and between them.^19,20^ For this reason, normative data is lacking for interpreting patient behavior; particularly given that what is considered appropriate affect varies considerably across settings and cultures. Evaluating affect from a phone call to tech support would require very different considerations compared to when an individual converses with a friend at a restaurant, responds to accusations by a police officer, or texts with a case manager. Moreover, affective expression occurs on a highly momentary scale, often seconds. Rating scales are not attuned to such changes and considerable information is potentially lost when ratings are based on observations over an extended period of time (minutes, hours). Hence, a clinical rating, even while seeking specificity, provides little information about when, where, and how affective expression is abnormal.^19^ For example, one cannot deconstruct a clinical rating to reveal that a patient, during a 30-min clinical interview, was diminished in expressing happiness and anger but exaggerated in expressing fear and sadness. Although the rating would likely be accurate in the hands of a skilled clinician the complexity and richness of the underlying observations would not necessarily be discernable from the anchor point selected. Similarly, clinical rating scales specifically targeting negative symptoms might have difficulty parsing out that a patient was relatively normal in the number, and duration of their facial expressions, but that the convergence of their expressions was dis-coordinated and ineffectual. Noting the disorganization could require a more comprehensive rating scale such as the PANSS where it would be scored on a different item. This type of sensitivity is critical for understanding potential mechanisms underlying diminished affect and for reducing mechanistic heterogeneity associated with them.

### Newer Approaches to Measuring Diminished Affect

Newer approaches to measuring affect involve objective behavioral analysis, including vocal acoustic, video facial, and movement analysis using wearable, mobile, and phone sensors. Although many of these technologies have existed, and have been used to understand psychopathology for decades, peer-reviewed publications employing them to understand negative symptoms have increased dramatically in the last decade. This, in part, reflects the explosion in the availability of inexpensive recording and sensing technologies, and at least two studies to date have employed audio and video from mobile phones to capture affect of clinically rated negative symptoms. A meta-analysis of studies employing acoustic analysis of patient speech has found that, in at least some studies, clinically rated negative symptoms are associated with less variability in pitch and volume and various other aspects of natural speech.^13,21^ Similarly, a handful of studies have demonstrated convergence between clinical ratings of negative symptoms and various features from computerized facial and head analysis.^22–25^ From this literature, it is well established that individuals with schizophrenia, particularly those with pronounced clinically rated negative symptoms, show deficits of facial expression of positive and prosocial emotions. It is also well established that clinically rated blunted affect is associated with *increased* facial expression of unpleasant emotions in many of these studies.^22–24^ Finally, it is fairly well established that acoustic features collected from speech sampling are inconsistent in their relation to clinically rated negative symptoms across studies.^19,20^

### Recommendations for Measuring Diminished Affect in a New Instrument

Implementing objective measures of affect provides unique opportunities for improving negative symptom assessment and could complement clinical decision-making by providing objective information about patients during clinical interviews. It can also potentially expand assessment by tracking patients while navigating their daily routines; eg, using audio/video diaries. In theory, these measures could provide detailed information about patients’ expressiveness; and this information could be processed and returned in near real-time. However, interpreting this information is a significant challenge given that affective expression in naturalistic environments is highly dynamic both within people and between them. While it is reasonable to evaluate an individual’s expressiveness using these technologies, it is not clear at this time how to determine whether this expressiveness constitutes a clinical concern or clinically relevant blunted affect. Developing automated solutions for interpreting these data is a complex, but eventually critical, endeavor.

### Specific Recommendations

Objective technologies can be used to quantify affective deficits from audio/video recordings. These technologies are more developed for detecting facial, rather than vocal or gestural expressions; though solutions for these have demonstrated proof of concept. As with alogia, blunted affect has primarily been assessed while interacting with a professional during a dyadic exchange. While it is unclear to what degree a clinical professional is needed for this, it does seem important that observations be collected from patients engaged in an activity that elicits prosocial behavior to a meaningful degree. For applications where exchanges with a live human are not feasible, a virtual agent might suffice. It will be important to optimize this agent, and the circumstances around interacting with it, such that prosocial behavior is elicited.

## Diminished Verbal Communication

### Current Assessment Methods

Verbal communication involves the use of systematic language to share information and is key in the clinical evaluation of negative symptoms. Measurement occurs across multiple components of language: including phonological, morphological, syntactical, semantic, and pragmatic levels, and there is evidence that abnormalities in verbal communication occur across each of these levels as a function of negative symptoms.^26,27^ For example, people with negative symptoms are more likely to have decreased speech production, use simpler and shorter phrases, make more syntactic errors, and use more word approximations and repetitions. Semantically, their spontaneously produced language tends to have fewer ideas, fewer emotion-related words, and more first-person singular pronouns.^26–28^ In clinical practice, these impairments are primarily detected through 3 methods: interactions with a trained professional during an unstructured mental status exam, standardized clinical interviews and rating scales, and standardized clinical neuropsychological tests (eg, verbal fluency tests) and performance-based (eg, Social Skills Performance Assessment) assessments. Collectively, these approaches offer face validity and acceptable psychometrics for their intended purposes.

### Limitations to Current Methods

There are key limitations to current methods. First, current methods require significant resources from both patients and researchers and generally require face-to-face interactions. Second, their ecological validity for understanding verbal communication is questionable, as it is unclear whether language samples elicited or measures used in research or clinical settings generalize to outside settings. Third, many current methods are agnostic to underlying mechanisms and unable to help disentangle whether anomalies reflect idiopathic (eg, cognitive, motivational) vs secondary (eg, depression, paranoia, medication side effects) causes. This is important as one considers that negative symptoms, and their response to interventions, are mechanistically heterogeneous. Fourth, most measures provide ordinal data with limited ranges that are insensitive to change, and hence, far from ideal for clinical trials.^29^ Fifth, it is unclear that current measures tap elements of verbal communication that are relevant to a patient. Traditional methods by-and-large are not capturing verbal communication examples from patients as they navigate their daily routine (eg, texting, calling a friend on the phone, and communicating by social media), which may be more central to a patient’s recovery. Sixth, many current methods are not deliberately attuned to cultural differences in communication, and hence, may be culturally insensitive for some applications.^30,31^ In sum, there is a need to bring greater sophistication, sensitivity, and objectivity to the measurement of verbal communication measures.

### Newer Approaches to Measuring Diminished Verbal Communication

There is a growing interest in using objective language and speech analysis to measure negative symptoms. This involves a suite of methods based on acoustic vocal analysis and Natural Language Processing (NLP) technologies. NLP approaches vary from basic text extraction and analyses (eg, text searches and word counts; identification of negative mood words), to highly sophisticated modeling from large corpora.^19,32–34^ In the past decade, significant advances have been made in large language modeling such that they can quantify aspects of verbal communication in fairly sophisticated ways. Proof of concept for measuring negative symptoms has been demonstrated in dozens of studies to date. For example, meta-analyses have reported relatively sizable relationships between clinically rated negative symptoms and increased pause times, fewer words being spoken, and slower speech rate.^13,21^ Clinically rated negative symptoms have also been associated with language anomalies more broadly, including reduced use of positive emotion and increased use of negative emotion words, reduced lexical diversity, reduced semantic complexity, fewer ideas, and unusual pronoun use.^26–28^ These relationships are observed across methods, eg, using in-person interviews and conversations, monologues, phone conversations, and performance-based tests.^22,32–34^ The use of machine learning to optimize the prediction of clinically rated negative symptoms has also been conducted in a handful of studies to date, with accuracy rates sometimes exceeding 90%.^35^ Other methods, such as using ambient acoustics and electronic health records mining, have also been recently employed, with encouraging preliminary findings.^36,37^

### Recommendations and Challenges for Measuring Diminished Verbal Communication in a New Instrument

There are several ways that new measures of verbal communication can likely contribute to negative symptom assessment. Speech from clinical interviews and interactions can be recorded and automatically processed in a way that could complement clinical decision-making, hence making it more comprehensive and potentially more efficient. Speech-to-text recognition systems that transcribe digitally recorded speech are widely available, inexpensive, highly accurate, developed for a wide range of languages, and can be applied to in-person clinical sessions as well as remote interviewing and telepsychiatry. A reasonable application is that these technologies could serve as a reference for clinicians evaluating the severity of alogia. With sufficient data, interpretive norms could be developed. Similarly, speech analysis can be paired with existing neuropsychological tests to help automate administration and complement verbal fluency, verbal memory and working memory, vocabulary and other verbal, and other language-related abilities. The advent of self-administered mobile neuropsychological testing, deployed using automated applications on smartphones, reflects an innovation from the last decade that can help expand the reach of negative symptom testing.^22^

There are other ways that novel verbal communication technologies could improve negative symptom assessment, though these are a bit more speculative. For example, “generative” AI (eg, ChatGPT, Bard, and Gemini) can be used to evaluate patient language, providing ratings that are similar, at least in structure, to clinical ratings based on existing rubrics (eg, from the BNSS). For example, a transcript of patient speech could be fed to a Gen AI system with a well-described rating rubric, comprehensive instructions, and exemplars. From this, GenAI could provide ratings resembling those from the CAINS or BNSS. The reliability and validity of this approach, to the best of our knowledge, have yet to be established. This could expedite the clinical evaluation of patients and expand language analysis beyond that observed in the clinical setting. Verbal technologies can also be used to develop avatars for automated administration.

However, there are challenges associated with implementing speech technologies for these applications. Very little is known about the reliability of verbal speech measures, and it is unclear the degree to which cross-sectional measurement picks up on aspects related to negative symptoms versus individual differences such as demographics, education level, global cognitive ability, and engagement.^22^ Given that negative symptoms are associated with a generalized deficit in schizophrenia, disentangling this issue is no small feat, and is particularly important if the objective technology will be used to capture change over short periods of time (eg, as an endpoint in a clinical trial). Moreover, individual language approaches are highly idiosyncratic, and the features employed in various studies are not identical or even comparable despite the use of similar names. A measure of “language complexity” may be derived using very different approaches and engineering parameters. A related challenge involves transparency and replicability across measures. While many simple NLP solutions employ transparent and replicable methods and technologies (eg, text searches using predefined word stems), online solutions using LLMs are often proprietary and inaccessible to clinicians, researchers, or regulators. Moreover, their development is increasingly being automated and governed by AI and thus these LLMs are constantly changing in a way that is difficult to audit. Hence, results can change dramatically over even a short period of time. Even if the algorithms underlying LLMs could be accessed and interpreted, they are constantly changing in such a way that complicates evaluation and regulation.^38^ Finally, there are many professional, legal, and privacy issues that have yet to be resolved or even realized. Applications with financial or legal consequences or that require regulatory oversight will likely take time to develop.

### Specific Recommendations for Measuring Diminished Verbal Communication in a New Instrument

Objective technologies have evolved sufficiently that they can be used to quantify verbal communication deficits and alogia from audio recordings. While the latter can be evaluated using structured texts and delineated topic speech samples, the former is primarily evaluated while interacting with a professional, eg, during a semistructured clinical interview. This is likely an important component of the assessment procedure, as the socio-cognitive demands placed on a patient may reveal alogia that may otherwise be less obvious. Objective alogia assessment can be used in complement to traditional “face-to-face” interviews. However, for applications where a live human interviewer is not available, a virtual agent should be considered. It is unclear whether a virtual agent can effectively reveal alogia in this regard; and as such, this remains an important question for future research.

## Diminished Sociality

### Current Assessment Methods

Current measures of diminished social interest are based upon structured interviews which rate the extent to which individuals desire, seek out, enjoy, initiate, and engage/persist in social activity with others.^11,14,39,40^ Ratings are based upon the report of the patient and often collateral information from an informant. Raters must ask sufficient questions to identify the nature of relationships and the interactions described. Raters must also examine the reasons behind limited socialization to ensure that negative symptoms rather than paranoia, social anxiety, or limited resources are the primary factors underlying the symptoms.

### Limitations of Current Methods

In all currently used interview measures of socialization for negative symptoms, the data are subject to recall bias on the part of the patient and any informant. All interviews rely on memory of events in a population in which memory has been shown to be impaired.^41^ The longer the recall period, the more likely it is that inaccuracies are present in the individual’s recall.

The definition of a social relationship can be in question. It is often unclear what is a “friend” as many interview participants list case managers and other treatment providers as their friends. Moreover, interview participants may view interactions with storekeepers and other casual acquaintances as social interactions because these can be the only individuals seen by the person during daily activity. Individuals being interviewed often appreciate the interactions with a case worker or store employee but these individuals would not reciprocally designate the patient as a friend.

Considering social media as a “social” activity also requires additional definition. Some recent studies suggest that higher social media use correlates with higher social isolation while other studies have suggested a relationship with paranoia.^42,43^ Different uses of media, such as scrolling through vs interacting on applications such as Facebook may represent different levels of socialization.

### Newer Approaches to Measuring Diminished Sociality

There are digital tools that are capable of examining some dimensions of socialization at a variety of different levels. EMA has been used to query participants about basic elements of social functioning, including asking if they are alone or with others and if what they are doing when with others involves what could be seen as a social engagement with the other person.^44,45^ Such assessments can be scheduled to occur multiple times during the day, over periods of weeks or months. While this approach can capture social context and the extent to which individuals are engaged in social behavior, the quality and depth of relationships would need to be assessed with additional queries, such as satisfaction with and perceived competence in the interaction or even the influence of concurrent mood states.^46,47^ The reciprocal nature of “friendship” remains hard to discern. EMA methods also query the dimension of desire for social relationships, studies have begun to examine social approach and avoidance motivation as well as querying whether a proximal interaction increased motivation to engage in the future.^48,49^ Specific queries could be developed to identify active social avoidance which is driven by mood states, social anxiety, suicidal ideation, or paranoia compared to negative symptoms. A potential variation of EMA is a structured assessment at the end of random days with questions regarding socialization. Patients can respond to written queries or avatars, recording their answers in either video or text format for later assessment. Ward et al demonstrated the effectiveness of using avatars in treating hallucinations in schizophrenia, suggesting that this approach can be applied to this population.^50^ This strategy would utilize face-to-face dialogue between the patient and a digital representation (avatar) of the interviewer. If successful it would eliminate the recall issue with infrequent in-office assessments and may produce data that more closely resembles the assessments currently used in clinical trials. The avatar would need to be programmed to use appropriate probes to uncover the necessary data. Scoring would still have to be completed later by a trained judge. The inclusion of the avatar in the process would be considered tentative pending validation against written prompts or a live interviewer.

Some passive methods of data collection such as geolocation could be used to identify the size of networks associated with a particular patient and map that network.^51,52^ Additional passive methods could be examining traffic by the individual on gaming and social media platforms, examining the number of text messages to friends, etc. Specific algorithms for understanding these data would need to be developed and tested. Rehki et al found that lower social media use was correlated with reduced socialization on the CAINS MAP asociality score.^40^ In addition to the methods identified above, ambient acoustics programs could “listen” at random times for the back and forth between the participant and another person as an indicator of conversation.

### Recommendations for Measuring Diminished Sociality in a New Instrument

Identifying the factors in diminished socialization that are the most indicative of negative symptoms and a digital assessment strategy that combines methods to get at these factors will take significant amounts of research. EMA and daily diaries at the end of the day are strategies that could be used almost immediately to assess this domain of functioning. Queries would need to be altered to identify reasons underlying problems with socializing including sadness, anxiety, and positive symptoms of psychosis. Some current interview-based approaches are designed to distinguish active from passive social withdrawal but may not do enough to eliminate social anxiety and other reasons for problems with socialization. An avatar or EMA could be configured to do this and, as noted above, the ability of EMA to capture current, antecedent, and consequential mood states can clarify these influences in a way that would be impossible to capture in any interview. Data regarding the relationship between socialization measures derived from EMA or an avatar interview would need to be compared to currently validated measures from negative symptom assessments. Both convergent and discriminant validity should be examined by comparing the novel measure to existing rating scales of negative symptoms and diminished socialization, as well as measures of mood states, fear, and paranoia, all of which contribute to the frequency of socialization. More passive measures such as ambient acoustics, examining social media and text activity, or geolocation would need much more time to be developed, standardized, and understood as indicators of sociality for a novel measure of negative symptoms. The use of these passive measures would hinge on research understanding their meaning in the context of negative symptoms of schizophrenia.

## Diminished Goal-Directed Behavior

Commonly referred to as avolition, amotivation, or apathy, reduced goal-directed behavior is a central feature of negative symptoms. Reductions are seen in social activities and productive activities including education and vocational engagement.^53^ As social activities and hedonic drives are described in separate sections, the focus here will be on the nature of reduced activities and their subjective correlates.

The observable behavioral symptoms of avolition include being home, alone, and engaging in sustained unproductive activities, including sitting, sleeping, resting, or doing nothing. The stereotype of the individual with negative symptoms sitting by themselves in front of the television for hours on end has been unfortunately confirmed with observational data collected with EMA.^54^

Consequences of reduced activity accrue though decrements in accomplishment in educational attainment and seeking and sustaining employment. In individuals whose symptoms develop during college, a common course is failure to return to school and gradual atrophy in interests, activities, and self-care to the point where they may require direction to perform basic tasks.

Other consequences of avolition include reductions in motivation to perform other tasks, such as shopping and cooking, leading to alterations in dietary habits such as subsisting on fast food.^55^ Also, reduced motivation is correlated with reductions in physical activity, such as walking or even standing, which can lead to health problems.^56^

Another important feature of avolition is that reduced motivation is defined as originating internally and is not due to reduced opportunities or moods. Another element of negative symptoms, lack of normal distress, commonly covaries with avolition.^9^ Someone with no friends, no money, and no daily activities, while completely untroubled by their situation, is the prototype. This lack of distress sustains avolition by eliminating the “negative reinforcement” motivation to control sadness or anxiety through positive activities.

### Current Assessment Methods

Interview-based strategies are used to assess avolition. As individuals with schizophrenia often struggle with awareness of their own behavior, contacting informants is a common practice.^57^ The quality of information coming from informants is quite variable, as described below. Further, many rating scales require normative considerations when generating ratings: “The ‘normal’ reference population against which the subject is to be compared is a young person in their twenties without schizophrenia” (NSA-16).^58^ The NSA excludes potentially bias-prone assessment strategies such as attempts at longitudinal comparisons within an individual, which are challenged by response biases and poor information. Subjective avolition can only be obtained by self-report, but the level of complexity of these judgments is reduced compared to making more complex judgments of competence, such as “How good are you at work-related tasks?” or subjective judgments of social competence. Finally, lack of normal distress is intrinsically a self-reported symptom. Rating scales targeting this feature simply query the level of subjective distress and index that to the level of potential distress-producing factors that exist in the individual’s life (unemployment, isolation, etc.).

### Limitations of Current Methods

Self-reports are challenging in schizophrenia and avolition is no exception. The primary origin of the challenge is not clear, but failures in recall, and response biases such as “hyperfocusing,” are certainly candidates.^59^ In the hyperfocusing case, the individual may have extraordinary performance in concentration, encoding, and recall of certain information (such as self-generated delusional ideation), but may not focus on other important elements of their situation. The result is that the participant cannot provide an accurate self-assessment of certain elements of their functioning because they never noticed it.

A major challenge resides in the requirement of some scales to rate competence. The less experience an individual has, the less information they will have to generate an accurate self-assessment. An individual who has never worked will have very limited information to estimate their vocational competence, with resulting responses based on a guess or a dominant response bias. For example, studies found that people with schizophrenia who had never been employed tend to rate their vocational abilities higher than those who are currently employed.^60^ Additionally, participants who consistently stay at home alone tend to rate their social abilities as superior compared to those who frequently spend time with others outside of the home.^61^

Certain informants, such as case managers or mental health technicians, who have regular contact with patients and focus on their functioning, provide functioning ratings that are remarkably well-correlated with indices of competencies.^62^ On the other hand, friend or noncaregiver relative informants provide information that can be uncorrelated with patients’ reports and performance.^63^ Given that many clinical trials requiring informants have quite liberal definitions of contact with participants, these strategies can be challenging.

### Newer Approaches to Measuring Diminished Goal-Directed Behavior

There are digital phenotyping strategies presenting alternatives to interview-based assessments, covering all elements of goal-directed behavior. These strategies include active surveys with EMA, which can sample location, social context, activities, and mood states, the intersection of which can parallel interview questions, but collected on a momentary basis. Passive strategies such as GPS can identify whether a participant is home or away, how long they are away when they leave, and can determine if someone is walking versus riding in a car, bus, or streetcar. Finally, smart band strategies can quantify activity, as well as sleep, thus providing a direct assessment of reduced activity and daytime sleep.

One of the appealing features of EMA is simplicity in the collection of outcome variables. Home vs away and alone vs with someone is a dichotomous quantification with direct relevance to goal-directed activities. This simple quantification also allows for the development of customized follow-up surveys that sample activities based on the contemporaneous intersection of home alone, home and with someone, or away. These surveys reduce the burden and likely increase cooperation by omitting irrelevant questions, such as counting social interactions when alone or asking about outside activities when at home.

Subjective elements of avolition are easily assessed with EMA, including some aspects that may not be transparent. It has been found that participants with bipolar depression currently engaging in unproductive activities report more sadness than when engaging in productive ones.^64^ In schizophrenia, the connection between sad moods and other elements of functioning measured with EMA seems weaker.^65^ However, querying activities first and moods second does not reveal that the questions are about connections between current moods and quality of activities. Participants who were home and alone for more than 90% of daytime surveys reported no sadness on these momentary surveys, suggesting a lack of distress.^66^ Another strategy to unobtrusively assess internal avolition is to follow surveys regarding observable goal-directed activity with queries regarding satisfaction with current activities or whether they would prefer to be doing something else.^49^

GPS and smart band passive data collection can also integrate with EMA.^67^ For example, validation of EMA responses with GPS technology is an appealing validity check. Further, an EMA report of being at the gym in the last hour when the smart band suggests 55 min of sleep is also informative regarding the validity of responses.

Table 2 presents the behavioral constellation associated with avolition as assessed by digital phenotyping strategies. Another substantial benefit of digital phenotyping is the collection of concurrent streams of activity and emotional experience. Because data are collected concurrently with repeated measurements, analysis of convergence (eg, moods and activities) is facilitated because the longitudinal course of individual variables can be correlated with the course of others measured at the same time. Time stamping of EMA surveys and passive measures allows for the integration of changing covariates across measurement strategies. Further, answers to current EMA surveys allow for qualitative interpretation of passive data to understand the goal-directedness of activities: Walking to the store vs pacing at home; exercising at a park vs wandering alone.

**Table 2.** Behavioral Constellation Associated With Avolition as Assessed by Digital Phenotyping Strategies

Home			
Alone			
Reduced total overall activities.			
	Only one activity reported in a survey		
Sustained nonproductive activities			
	Sitting alone		
	Sleeping		
	Resting		
	Nothing		
		– Defining sustained	
			Across multiple surveys
			Similar number and quality of activities
Failure to socialize when others are present.			
			No differences in activities when alone or with others

### Methodological Considerations Relevant to Measurement of Diminished Volition: Adherence

Any new technological method will require careful development and validation, including adherence, and addressing avolition presents its own set of challenges. It is reasonable to question whether an individual who manifests significant avolition will respond to queries. This question is addressed conceptually by the results of previous studies: An individual who answers 85/90 surveys over 30 days, always home and alone, reporting a single activity at each survey, most commonly watching television, clearly appears to manifest avolition.^68^ However, that determination is based on the answers to 94% of 90 EMA surveys over a month.

Some issues associated with active methods have been addressed, including survey frequency, study duration, and interference from other symptoms.^69^ Adherence did not differ when participants with schizophrenia were surveyed 7 times per day vs 3 times per day.^70^ Adherence did not decline over a 30-day survey period and baseline severity of psychotic symptoms had a correlation of zero with 30-day survey adherence. Adherence to passive strategies has been examined as well, with the results suggesting very suitable adherence to these strategies.^71^

It is important to note that these data come from studies where participants were compensated for completing surveys and were notified of their compensation immediately after each survey they completed. Research suggests that lack of compensation is associated with markedly lower adherence. Furthermore, providing cumulative or weekly compensation, as opposed to notifying participants about their compensation after each survey, is associated with reduced adherence.^72^ Studies also indicate that multiple surveys per day lead to higher adherence compared to single daily surveys and that using predictable and consecutive daily survey strategies (5–7 days per week) seems to yield better adherence than episodic strategies.^72^

### Recommendations for Measuring Diminished Goal-Directed Behavior in a New Instrument

There are several critical recommendations for a new assessment instrument. Behaviors reflecting reduced motivation should be examined with both active and passive digital phenotyping strategies. Location and social context can be triangulated with EMA surveys and passive measurement because being home and alone for the majority of a sampling period is commonly a reflection of reduced motivation. Socially and functionally relevant activities need to be examined across home and away locations, acknowledging that it is entirely possible to engage in productive and social activities at home. Activities need to be characterized as productive/social vs unproductive and the dimension of physically active vs inactive needs to be extracted from these EMA surveys. Interpreting passive measures such as step counts or away-from-home travel requires a concurrent description of activities to differentiate purposeful activity from pacing or wandering. Subjective avolition has to be examined, which is likely to be assessed on a post hoc basis through the combination of queries regarding activities planned for the future as well as an assessment of the subjective evaluation of recently completed activities, as described in the section on reduced hedonic drives. Assessment of mood state variables, including anxiety and depression, are central requirements for viewing reduced goal-directed behavior as a direct consequence of schizophrenia and not a secondary, and likely more transient and variable, feature of mood states.^53^

## Diminished Hedonic Drives

### Current Assessment Methods

There is 5-decade history of scales to measure “anhedonia” which continues to be referred to as a cardinal symptom of schizophrenia.^65^ In reviews describing and critiquing both the older and more recent scales none emerge as a “gold standard” on which there is alignment.^73–75^ Moreover, it is now appreciated that at least in schizophrenia, the term “anhedonia” is highly complex, and many of its components, such as in-the-moment experience, are not impaired. Thus, trials aimed at negative symptoms in schizophrenia are not aimed at anhedonia per se which is being pursued, for instance, in trials that target apathy in Alzheimer’s disease.^76^ In patients enrolled in these studies, the absence of experiencing pleasure from normally pleasurable activities and stimuli is part of the diagnosis. Something as straightforward as the apathy subscale of the Neuropsychiatric Inventory (NPI) can be affected by treatment in a positive direction.^68^ How this scale or its subscales developed for neurodegenerative disorders would perform in schizophrenia is an open question and only mentioned here because similar terms are being used for symptoms across disorders for which separate scales have been developed.

In a recent article, Marder and Umbricht emphasize the potential importance of alternate scales and measures to get at what is conceptualized as the anhedonia-amotivation dimension of negative symptoms such as the BNSS and Clinical Assessment Interview of Negative Symptoms (CAINS).^1^ From the perspective of scales used in trials targeted at negative symptoms, however, it is the Marder subscale of the PANSS that is most often utilized. Other studies have incorporated the CAINS, the BNSS, or a derivative of the PANSS using an approach, the uncorrelated PANSS score matrix, to deal with partial correlations of negative and nonnegative factors.^1^ Given that many of the older scales and even some more recent ones are either not being used or are infrequently used in trials they cannot be classified as “current.”

### Limitations of Current Methods

The terminology used when moving from a diagnosis of negative symptoms to scales reveals why subjective scales are likely to suffer from multiple limitations. Moving from earlier results of factor analysis of 5 diagnostic symptoms (blunted affect, alogia, avolition, asociality, and anhedonia), even when grouped into 2 subdimensions called “diminished expression and avolition/apathy” to severity scales raises the challenge of quantifying cultural and context-dependent terms. A major approach to linking what humans express to the constructs implicit in terms such as anhedonia is the Research Domain Criteria (RDoC) research framework. It falls most directly under the broadly conceptualized positive valence systems domain but can be easily seen as influenced by the negative valence domain (eg, fear that a reward will not be forthcoming) or some aspect of the cognitive domain whereby what is learned and remembered from experience (which might be faulty in schizophrenia and other psychiatric disorders) has a sustained effect on a drive. Moran et al discuss in detail a model of how having a pleasurable experience (“reward”) links to motivated behavior which is impaired in schizophrenia.^73^ One component of the model, “reward anticipation,” is viewed as something that can be evaluated with psychological testing paradigms that go beyond current scales and has been argued by some to be closer to what is altered in the hedonic realm in schizophrenics. However, the testing paradigms required are not viewed as practical for clinical trials.

A few years ago NIMH formally solicited research proposals targeting approaches to understanding hedonic responses in preclinical studies with the hope that these might generate some novel translational possibilities. It is too early to know what may emerge, leaving the field with classic induction and reversal of anhedonia, especially in rats, as the preclinical reference point for generating models of the components of hedonic experience in humans. This is to recognize that the neuroscience-aligned concepts underlying the RDoC conceptualization have not yet produced data useful for developing better clinical scales beyond decades-old ways of inducing anhedonia in rodents.

Proposals taking into account preclinical research as to what might be of greatest value relevant to negative symptoms focus on one or more of the components delineated in models of seeking a hedonic experience such as reward seeking.^77^ To some extent the preclinical paradigms do have analogs in humans using fMRI and EEG paradigms (like the Monetary Incentive Delay task), a point to which we will return. To appreciate the complexity involved in dissecting components proposed as part of what is involved in hedonic responses in schizophrenia one can consider the approaches and challenges involved in quantifying “effort valuation”—basically, is the effort required to obtain a certain level of reward worth it?—including various psychological paradigms coupled to fMRI measures.^78^ Beyond the limitation of the methods involved there remains uncertainty as to whether such components of the most current psychological clinical models of hedonic experience are valid in terms of linking to some ultimately discoverable brain process. One could identify a subset of patients with negative symptoms of schizophrenia who showed a quantifiable alteration of an fMRI signal to a reward-related task and enter them into a trial to see if an intervention firstly “improved” that signal and only then, secondly, assess whether this related to a clinical measure related to anhedonia as has been done for a kappa receptor opiate antagonist in depressed or anxious patients with anhedonia.^79^ Something like this would be needed to provide a case for such complex approaches to exploring novel treatments for negative symptoms.

And in the real world of patients likely to be considered for clinical trials, there is confound of both illicit and prescription drug use. Psychological states and cognitive processes can be influenced by both “rewarding” compounds such as cocaine and impairing components of some antipsychotics. The extent to which such factors have been adequately controlled in studies on the hedonic aspect of negative symptoms is not clear but is likely relevant to many of the mixed findings noted in a recent review of the field.^73^

### Newer Approaches to Measurement of Diminished Hedonic Drives

It is hoped that components of the hedonic drive/experience in schizophrenia such as anticipatory pleasure, effort valuation, and encoding/memory for pleasurable experiences may prove usefully accessed by digital measures like EMA in daily life without relying on complex testing paradigms as proposed in a recent review.^73^ Passive and especially EMA remote measures offer other possibilities for capturing a range of parameters in an observer or interviewer unbiased quantitative manner. These allow for a much greater frequency of assessments than is possible with traditional rating methods which might allow for more sensitive detection of drug effects. One could argue that the type of actions and activities captured by passive measures, including unprompted speech, at the very least would establish, using an individual as his or her own control measured over a long enough period before treatment, whether there was a general shift in whatever behavior was being measured. If there were a way of ascertaining whether such a shift was purposeful then that might be argued to quantify the degree to which an individual is driven to do something.

With EMA, in addition to the range of questions that might be asked one could, if requiring a voice response, add another level of inquiry that might be relevant to the quantification of “drive.” What is not yet clear is whether EMA responses to probes of one or more components of hedonic experience will show either the kind of psychometric properties needed or tap into anything that will be more sensitive to a drug effect than more global and traditional measures. Existing paradigms for assessing such constructs as reward evaluation may not be appropriate for people with schizophrenia given that living with the illness might well be associated with very different valuations of what is rewarding than typical of populations used to develop the testing paradigms. It seems very unlikely that there is a universal set of questions that get at the range of constructs discussed in the literature. Another approach is to build a unique set of questions for each individual, such as has been done with previous anhedonia measures (eg, The Dimensional Anhedonia Ratings Scale; DARS: Rizvi et al.).^80^ This strategy would be challenging to implement in a clinical trial environment, based on regulatory concerns about the similarity of data collected across individuals. Nonetheless, this might be the most sensitive way of detecting change in an individual but raises the question of how to combine change data across individuals. The idea of using individualized measures is an old one that has not yet demonstrated advantages over traditional measures in sensitivity to the detection of drug effects. Nonetheless, conceptually it remains an attractive possibility to explore.

There have been some studies to date which are summarized in table 3 that pilot remote measures focused on aspects of hedonic experience. Overall, these studies indicate that remote measures may capture some of what is addressed with direct patient interviews along with a least one surprising finding of patients reporting *higher* anticipated pleasure from activities than normal using a phone-based interview method.^66^ The relationship between complex in-lab assessments of, for instance, how much cognitive effort a patient will spend for a certain degree of reward and EMA assessments is not, however, that clear (see table 3).

**Table 3.** EMA Studies Directly Relevant to Assessing Anticipatory Pleasure in Schizophrenia

Study		Supports Anticipatory Pleasure Deficit in SCZ?	Subjects				Device			Frequency			Assessment				EMA Questions			Main Results			Other Findings
Gard et al^81^		Yes	Patients (SCZ, SAD = 15); healthy: 12				Pagers and booklets			7 pages per day for 7 d			Participants wrote down what they were doing and rated amount of enjoyment they were experiencing (0–5 scale); participants wrote down what they were looking forward to doing and the enjoyment they anticipated experiencing. Current and looked-forward-to activities were chosen from a list of 15 activities. Activities were coded as “goal-directed” or “nongoal directed”				Anticipatory pleasure ratings, consummatory pleasure ratings (0–5 scales)			Patients less frequently engaged in, looked forward to, and expected pleasure from goal-directed activities than controls, but the groups did not differ for engaging in and looking forward to nongoal related activities. No differences in anticipatory pleasure for nongoal directed activities. No differences in consummatory pleasure for goal- directed and nongoal-directed activities			
Gard et al ^74^		No	Patients (SCZ, SAD = 47); healthy: 41				Cell phone calls			4 calls per day for 7 d			Interviewer calls and asks semistructured questions about: (1) current environmental context; (2) number of upcoming goals; (3) anticipatory pleasure for upcoming goals; (4) expected difficulty of upcoming goals; (5) completion of goals; (6) experienced pleasure for completed goals. All current activities and goals were independently coded in terms of “pleasure-based,” “effortful,” “long-term benefit” (0–3 scales)				Anticipatory pleasure ratings, consummatory pleasure ratings, anticipated difficulty ratings all on 0 (not at all) to 5 (extremely) scales			Despite a similar number of goals and activities between groups, patients had less effortful goals and activities and fewer long-term benefit goals than healthy participants. No differences in consummatory pleasure. Unexpected finding: patients reported *higher* anticipated pleasure of goals than normals, and reported *more* goals that were more pleasure-based than normals (possibly associated with method of calling patients)			Trend for patients to anticipate social activities as less enjoyable. Some modest associations with cognition, negative symptoms, and employment status.
Edwards et al^82^		No	Patients: 36; healthy: 44				PDA device			7 prompts per day for 6 d			Activity (current and future); Mood (4 positive emotion ratings, 7 negative emotion ratings); Anticipatory and consummatory pleasure ratings (0–7); Estimate of chance of completing activity (0–100%); Motivation and expectation (rating of interest on 0–7; rating of rather do something else on 0–7); Current and future social pleasure; Preference to be alone/with others				Various 0–7 scales			Anticipatory pleasure was higher for patients for both functional and leisure activates than for controls. Expectation of completing functional activities was lower for patients than controls. No differences in anticipatory pleasure for social activities. Patients preferred to be alone more than controls.			Similar amount of leisure activities between groups, but less functional and more nothing/resting activates in patients.
Shovestul et al^83^		No	Patients (SCZ/SAD); 34; healthy: 43				Internet-based diary. Daily email reminders prompted subjects to complete the diary.			1 diary per day before bed for 8 d			Focused on “meaningful social interactions.” Report all anticipated social interactions in the next 24 h. Provide anticipated emotion ratings (for 13 different emotions; 6 positive, 7 negative emotions) for all anticipated interactions. Report whether they actually engaged in anticipated interactions from the prior day. Provide 13 emotion ratings for all completed interactions.				Emotion ratings on a 0–5 scale			Average affective forecasts overall were less accurate in patients than controls. This was accounted for by less accurate predictions for negative emotions; there was no group difference for positive emotions.			Self-report ratings on trait social anhedonia and social pleasure significantly correlated with forecasting accuracy for negative emotions. There were no correlations for accuracy of positive emotions. Much of data collected during COVID social distancing.
Indirectly relevant studies																							
Study	Supportive?				Subjects			Device			Frequency			Assessment		EMA questions			Main results			Other findings	
Brenner and Ben-Zeev^84^	No: patients over-report anticipated pleasure compared to experienced pleasure (but no control group)				Patients (SCZ, SAD): 24			PDA device			6 prompts per day for 7 d			Completed affective forecasting measure (for 8 different emotions over the coming week) in lab prior to EMA. EMA assessment had patients rate emotional experience for the same 8 emotions.		Ratings of 8 emotions (0–4)			Patients overestimated intensity of all (3/3) positive emotions experienced and most (3/5) negative emotions experienced. Overestimate of positive emotions was larger than overestimate of negative emotions.			Ben-Zeev et al. ^84^ found that patients overestimate their retrospective recall of affective experience	
Moran et al^85^	Yes: general support for convergent validity between EMA and negative symptom ratings of pleasure/motivation				Patients (SCZ, SAD): 34			Smartphone EMA			4 prompts per day for 7 d			Questionnaire modeled on the CAINS Motivation and Pleasure (MAP) subscale; current activities; motivation for reported activities (0-5 rating); pleasure in reported activities (0-5 rating); motivation and pleasure ratings because last beep; motivation and pleasure ratings for upcoming 2-3 h		Ratings of past, current, and expected motivation and pleasure on 0–5 scales			Good convergent validity between CAINS and EMA assessments of motivation and pleasure. Did not include a control group to evaluate whether patients showed a deficit in anticipatory pleasure ratings.			EMA ratings of motivation and pleasure associated with reinforcement learning and effort decision-making laboratory tasks; EMA related to working memory but not premorbid IQ	
Culbreth et al. ^86^	No: minimal support for convergent validity between laboratory decision-making task and EMA assessment of pleasure/motivation				Patients (SCZ, SAD): 28			Smartphone EMA			4 prompts per day for 7 d			Current activities (from the list), level of interest, level of enjoyment; Past activities, interest, enjoyment because last prompt; Future activities, interest, enjoyment expected in next 2–3 h.		Various 1–5 scales			No significant correlation between EMA and performance on an effort-based decision-making task (though there were some trends). No EMA data collected in the control sample in this study to evaluate whether patients show an anticipatory pleasure deficit.				
Distantly related studies																							
Study	Supportive?			Subjects		Device			Frequency			Assessment			EMA questions			Main results			Other findings		
Sanchez et al^87^	N/A			Patients (SCZ, SAD): 47; healthy: 41; overlapping sample with Gard et al		Cell phone calls			Four calls per day for 7 d			Interviewer calls and asks semistructured questions about (1) current positive state; (2) current negative emotional state; (3) current activities; (4) enjoyment of current activity			Ratings of specific emotions (0–5 scales) summarized into positive and negative measures; enjoyment rating (0–5)			This study is not directly relevant to anticipatory pleasure or effort. No group differences in positive emotion or enjoyment from activities, but patients reported more negative emotions. Weaker (negative) correlation between negative emotion and enjoyment of activities in patients than in controls—ie, patients feel more negative emotion during pleasant events than controls			No correlations between positive emotion or enjoyment with clinical symptoms; negative emotions are correlated with depression. Minimal correlations with neurocognition.		
Granholm et al.^88^	N/A			Patients (SCZ): 100; healthy: 71		Smartphone EMA			7 prompts per day for 7 d			Time spent at home and in functioning behaviors during the past hour, number of social interactions/social context. Nonproductive activities in past hour.			Focused only on number of different types of activities (ie, no Likert scale ratings or pleasure, interest, effort)			Not directly relevant. Patients spent more time at home engaged in fewer productive activities in or out of home leisure, reported more nonproductive activities, less frequently transported independently. No significant differences for productive self-care or home-care activities. Patients reported fewer social interactions.			EMA showed good correspondence with objective indicators of functioning, and significant/modest correspondence with lab measures/sales of functional capacity/functioning; Related EMA results from this data set by Strassnig et al^54^; Depp et al^65^		
Merchant et al^89^	N/A			Patients (SCZ, SAD): 66		Smartphone EMA			4 prompts per day for 7 d			Indicate current, past, and anticipated activities on a drop-down list; activities characterized as goal-directed or nongoal directed; planned vs completed (ie, at least one completed) goals were evaluated			Focused only on number of activities planned and completed (ie, no Likert scale ratings or pleasure, interest, effort)			Not directly relevant. Patients planned fewer goal-directed activities than they completed; MAP scores on the CAINS correlated with planned goal-directed behavior on EMA; performance on an effort-based decision-making laboratory task (EFFrT) correlated with planned and completed goal-directed activities on EMA			Working memory did not significantly correlate with EMA; UPSA-B did correlate with EMA; results were not accounted for by depression		

Most of the findings that have emerged from the remote measures tabulated in table 3 are consistent with interview-based ones without it being clear that eliciting the information in this manner will prove more or less sensitive in detecting change than the standard scales as applied in clinical trials to date that target negative symptoms. The studies do support that over a 7-day period patients respond at high rates but no studies address the question of what would happen in 6- to 12-week (or longer) trials. Moreover, because only one of the studies explicitly compares negative symptom ratings (using the CAINS) with EMA responses the issue of whether a negative symptom subgroup would comply as well as the broader population is not addressed. In that study, there were only 5 patients with high CAINS ratings with the main finding being a relationship to degree of effort in the laboratory effort expenditure task.^77^ There was no relationship between the formal lab test and EMA measures. Overall, the clearest findings from the remote measures studied were to document various forms of altered valuation and degrees of activities without showing that one or another related closely to baseline measures of negative symptoms. Changes in activity associated with drug treatment could, nonetheless, be useful as a means of signal detection whether or not it tracked closely with standard ratings of negative symptoms.

### Recommendations for Measuring Diminished Hedonic Drives in a New Instrument

Out of one or more remote assessments of the types elaborated in some detail in other sections of this article, one might then develop a new operational definition for an aspect of hedonic drive that is relevant to schizophrenia. The FDA and the field are sensitive to the issue that when we use words like anhedonia and apathy they may mean very different things in different disorders so having a disorder-specific measure still makes sense for a domain that cuts across populations. As part of any such process, generating normative data using the same remote measures might prove important so that remote measure data from clinical trials could be compared with other measures in a normal population related to health and function. Based on remote data generated to date it is uncertain whether current EMA questions or probes will capture some of what is detectable with in-person testing paradigms.

The field could invest in an EMA-based approach specific to a component of the model of hedonic experience summarized by Moran et al (2022) in an article arguing for exploration of EMA measures.^73^ As an example, it might be possible to find novel probes for reward anticipation and see if an EMA-based measure and a lab-based one produced comparable findings in an individual. If so, this would argue for the new EMA measure to be deployed into clinical trials. Given the variability in results with the lab-based measure, one might even still explore the EMA measure in a trial if it had reasonable psychometric properties.

## Discussion

There was a consensus among the authors that the current approaches to measuring negative symptoms in clinical trials are flawed. Although newer clinical interview-based assessments explicitly address all 5 domains of negative symptoms they share important limitations: They require an interviewer to rate relatively subtle phenomena such as diminished expressiveness and speech; they rely on a subject’s ability to recall their interests and feelings over a past period such as a week; and they assume that a subject feels and behaves similarly in a clinical setting and during their regular everyday life. These limitations can be addressed by alternative methods that are in different stages of development. That is, newer remote digital assessment methods can measure relatively subtle phenomena with greater accuracy and they can measure thoughts and feelings at random times during the patient’s regular life routines eliminating the need for recall. Modifications of these remote measurement technologies are also applicable to measuring the behavioral impact of treatment on a variety of CNS and nonCNS therapeutic areas beyond schizophrenia.

The process for developing a multimodal instrument that can improve the measurement of negative symptoms should focus on those that are believed to be sufficiently developed to be included in the instrument and those that are practical for large clinical trials. As a result, measures using artificial intelligence (AI) to measure speech or measures that would use EEG or fMRI will not be included. It is reassuring that all the methods that are ready for further evaluation collect data with a smartphone. Unlike current clinician-administered negative symptom assessments these approaches provide far more sampling points, assess the patient in their own environment as they go about daily life, and are not dependent on accurate patient and caregiver recall of events and mood states. The new instrument would assess behaviors reflecting reduced sociality, goal-directed behavior, and hedonic drives with the active (eg, EMA) and passive smart band-based (eg, GPS, and actigraphy) digital phenotyping strategies discussed earlier in this article. Location and context could be triangulated with time-stamped EMA surveys and other passive measurements, longitudinally, allowing for the integration of changing covariates (eg, activity, mood, context) across measurement strategies. Verbal communication deficits and alogia would be assessed from audio recordings using structured texts and delineated topic speech samples in the context of a semistructured clinical interview. The latter could be conducted by a professional, virtual agent, or avatar. The diminished affect will be assessed by vocal acoustic, video facial, hand, and body movement analysis using wearable, mobile, and phone sensors.

The process for developing an instrument could resemble the NIMH MATRICS process that was used to develop a battery for measuring cognition in schizophrenia.^90^ The process included a number of steps including selecting the domains of cognition that should be measured; agreeing on the criteria for the selection of a measure which can include its psychometric characteristics, its feasibility for inclusion in clinical trials, and the evidence that the measure is a valid tool for measuring the domain. Using these criteria, a group of experts selected the instruments that would be included in a beta version of the battery. The beta version was then assessed in a psychometric study that included a study for the development of norms. Other processes included the use of a nonprofit entity to package the instrument and develop agreements with the owners of tests and the translation of the battery into a number of languages.

There are several serious challenges in developing a multimodal negative symptom instrument. Perhaps the most serious is determining how to validate a candidate measure given that the current clinical measures are highly suspect. Nevertheless, consistent with findings presented in the domain sections of this article the phenomena measured by the multimodal instrument could be predicted to overlap with the findings of existing clinician-administered negative symptom instruments and exhibit a significant correlation. However, interpretation of convergent validity with scales such as the BNSS, CAINS, NSA-16, or Marder Negative factor would be caveated due to the more frequent sampling and objectivity of digital measures versus the dependency on recollection by the subject, sampling bias, and subjectivity of the clinician-administered scales. Discriminant validity can be examined by comparing the novel measure to measures of mood states, fear, paranoia, and extrapyramidal side effects. The temporal stability of the new instrument can be assessed in stable patients. The relationship of the instrument to function and clinical meaningfulness of change can be assessed in comparison with global and quality of life measures as well as with virtual and real-world measures of function. Patients, caregivers, and clinicians should be directly queried on their views of the content validity of the instrument and in defining minimal clinically significant change. The relationship among cognitive impairment, avolition, and anhedonia should be evaluated. Normative data can be collected in different populations to assess for cultural differences in domains such as expressiveness and social behaviors.

The video analysis of facial expressions from video; the analysis of audio recordings for expressiveness and verbal content; and the analysis of GPS and actigraphy data will require the use of sophisticated software. A method for incorporating this method of analysis into the multimodal battery will need to be developed. It is also unclear whether or not a clinician rating should be included in the multimodal battery.

Neither existing clinician-administered scales nor currently conceived remote measurement scenarios directly address the distinction between primary and secondary negative symptoms. The identification of negative symptoms stemming from depression, positive symptoms, social anxiety, or iatrogenic causes (eg, antipsychotic-induced akinetic Parkinsonism or sedation), eg, has important treatment implications. Questions about mood, anxiety, and psychotic symptoms embedded in EMA could be informative in this regard. It is conceivable that machine learning applied to voice analysis and/or actigraphy, eg, could eventually distinguish some patterns more commonly associated with primary vs secondary negative symptoms.

Obtaining high levels of compliance with study procedures from subjects with more severe motivational deficits is another challenge in remote data collection. As discussed earlier in this article, examples of practices associated with high levels of adherence include financial incentivization and frequent correspondence with the patient.^69–72^

Patients, caregivers, and other relevant stakeholders will be involved in the design of the instrument and asked to provide feedback on the data collection procedure to assess usability, clinical relevance, and safety or confidentiality concerns. The process will require very close attention to addressing any privacy concerns that cannot be addressed by password or biometric authentication. Confidentiality during the collection of data for voice and facial analysis and during interaction with avatars may be challenging to obtain and require private spaces or technical enhancements during day-to-day activities.

The rapid growth and scope of technologies applicable to remote evaluation of negative symptoms necessitates that the development of a multimodal instrument be an iterative process subject to ongoing revision. Not all promising approaches have been addressed in this publication.

In summary, the authors agreed that there is a compelling need for a method for assessing negative symptoms that does not involve solely clinical ratings. There is also agreement that a measure should be multimodal and include data that can be collected with a smartphone or a wearable. Although the development of such an instrument faces substantial challenges, none of them are insurmountable.

## References

[1] Marder SR , Umbricht D. Negative symptoms in schizophrenia: newly emerging measurements, pathways, and treatments. Schizophr Res. 2023;258:71–77. doi:10.1016/j.schres.2023.07.010 37517366

[2] Kirkpatrick B , Fenton WS, Carpenter WT, Marder SR. The NIMH-MATRICS consensus statement on negative symptoms. Schizophr Bull. 2006;32(2):214–219. doi:10.1093/schbul/sbj053 16481659 PMC2632223

[3] Daniel DG , Cohen AS, Velligan D, et al Remote assessment of negative symptoms of schizophrenia. Schizophr Bull Open. 2023;4(1):sgad001. doi:10.1093/schizbullopen/sgad001 39145343 PMC11207840

[4] Daniel DG. Issues in selection of instruments to measure negative symptoms. Schizophr Res. 2013;150(2-3):343–345. doi:10.1016/j.schres.2013.07.005 23899996

[5] Weigel L , Wehr S, Galderisi S, et al The Brief Negative Symptom Scale (BNSS): a systematic review of measurement properties. Schizophrenia. 2023;9(1):45. doi:10.1038/s41537-023-00380-x 37500628 PMC10374652

[6] Wehr S , Weigel L, Davis J, Galderisi S, Mucci A, Leucht S. Clinical Assessment Interview for Negative Symptoms (CAINS): a systematic review of measurement properties. Schizophr Bull. 2023;50:747–756. doi:10.1093/schbul/sbad137 PMC11283189 37951838

[7] Starzer M , Hansen HG, Hjorthøj C, Albert N, Nordentoft M, Madsen T. 20-year trajectories of positive and negative symptoms after the first psychotic episode in patients with schizophrenia spectrum disorder: results from the OPUS study. World Psychiatry. 2023;22(3):424–432. doi:10.1002/wps.21121 37713547 PMC10503930

[8] Ang MS , Rekhi G, Lee J. Validation of the Brief Negative Symptom Scale and its association with functioning. Schizophr Res. 2019;208:97–104. 30987926 10.1016/j.schres.2019.04.005

[9] Strauss GP , Keller WR, Buchanan RW, et al Next-generation negative symptom assessment for clinical trials: validation of the Brief Negative Symptom Scale. Schizophr Res. 2012;142(1–3):88–92. doi:10.1016/j.schres.2012.10.012 23127378 PMC3502630

[10] Blanchard JJ , Bradshaw KR, Garcia CP, et al Examining the reliability and validity of the Clinical Assessment Interview for Negative Symptoms within the Management of Schizophrenia in Clinical Practice (MOSAIC) multisite national study. Schizophr Res. 2017;185:137–143. doi:10.1016/j.schres.2017.01.011 28087270

[11] Kring AM , Gur RE, Blanchard JJ, Horan WP, Reise SP. The Clinical Assessment Interview for Negative Symptoms (CAINS): final development and validation. Am J Psychiatry. 2013;170(2):165–172. doi:10.1176/appi.ajp.2012.12010109 23377637 PMC3785242

[12] American Psychiatric Association. Diagnostic and Statistical Manual of Mental Disorders. 5th ed. Washington, DC USA: American Psychiatric Association; 2013. doi:10.1176/appi.books.9780890425596

[13] Cohen AS , Mitchell KR, Elvevåg B. What do we really know about blunted vocal affect and alogia? A meta-analysis of objective assessments. Schizophr Res. 2014;159(2-3):533–538. doi:10.1016/j.schres.2014.09.013 25261880 PMC4254038

[14] Kirkpatrick B , Strauss GP, Nguyen L, et al The Brief Negative Symptom Scale: psychometric properties. Schizophr Bull. 2011;37(2):300–305. doi:10.1093/schbul/sbq059 20558531 PMC3044634

[15] Horan WP , Kring AM, Gur RE, Reise SP, Blanchard JJ. Development and psychometric validation of the Clinical Assessment Interview for Negative Symptoms (CAINS). Schizophr Res. 2011;132(2-3):140–145. doi:10.1016/j.schres.2011.06.030 21798716 PMC3196271

[16] Dobbs JL , Sloan DM, Karpinski A. A psychometric investigation of two self-report measures of emotional expressivity. Pers Individ Differ 2007;43(4):693–702.

[17] Cohen AS , Matthews RA, Najolia GM, Brown LA. Toward a more psychometrically sound brief measure of schizotypal traits: introducing the SPQ-Brief revised. J Pers Disord. 2010;24(4):516–537. 20695810 10.1521/pedi.2010.24.4.516

[18] Loewy RL , Bearden CE, Johnson JK, Raine A, Cannon TD. The prodromal questionnaire (PQ): preliminary validation of a self-report screening measure for prodromal and psychotic syndromes. Schizophr Res. 2005;79(1):117–125. 16276559

[19] Cohen AS , Schwartz E, Le TP, et al Digital phenotyping of negative symptoms: the relationship to clinician ratings. Schizophr Bull. 2021;47(1):44–53. doi:10.1093/schbul/sbaa065 32467967 PMC7825094

[20] Cohen AS , Cox CR, Masucci MD, et al Digital phenotyping using multimodal data. Curr Behav Neurosci Rep. 2020;7(4):212–220. doi:10.1007/s40473-020-00215-4

[21] Parola A , Simonsen A, Bliksted V, Fusaroli R. Voice patterns in schizophrenia: a systematic review and Bayesian meta-analysis. Schizophr Res. 2020;216:24–40. doi:10.1016/j.schres.2019.11.031 31839552

[22] Cohen AS , Cowan T, Le TP, et al Ambulatory digital phenotyping of blunted affect and alogia using objective facial and vocal analysis: proof of concept. Schizophr Res. 2020;220:141–146. doi:10.1016/j.schres.2020.03.043 32247747 PMC7306442

[23] Abbas A , Hansen BJ, Koesmahargyo V, et al Facial and vocal markers of schizophrenia measured using remote smartphone assessments: observational study. JMIR Form Res. 2022;6(1):e26276. doi:10.2196/26276 35060906 PMC8817208

[24] Cowan T , Masucci MD, Gupta T, Haase CM, Strauss GP, Cohen AS. Computerized analysis of facial expressions in serious mental illness. Schizophr Res. 2022;241:44–51. doi:10.1016/j.schres.2021.12.026 35074531 PMC8978090

[25] Gupta T , Haase CM, Strauss GP, Cohen AS, Ricard JR, Mittal VA. Alterations in facial expressions of emotion: determining the promise of ultrathin slicing approaches and comparing human and automated coding methods in psychosis risk. Emotion. 2022;22(4):714–724. doi:10.1037/emo0000819 32584067 PMC7759595

[26] Kuperberg GR. Language in schizophrenia part 1: an introduction. Lang Linguist Compass 2010;4(8):576–589. doi:10.1111/j.1749-818X.2010.00216.x 20936080 PMC2950318

[27] Kuperberg GR. Language in schizophrenia part 2: What can psycholinguistics bring to the study of schizophrenia…and vice versa? Lang Linguist Compass. 2010;4(8):590–604. doi:10.1111/j.1749-818X.2010.00217.x 20824153 PMC2932455

[28] Covington MA , He C, Brown C, et al Schizophrenia and the structure of language: the linguist’s view. Schizophr Res. 2005;77(1):85–98. doi:10.1016/j.schres.2005.01.016 16005388

[29] Fusar-Poli P , Papanastasiou E, Stahl D, et al Treatments of negative symptoms in schizophrenia: meta-analysis of 168 randomized placebo-controlled trials. Schizophr Bull. 2015;41(4):892–899. doi:10.1093/schbul/sbu170 25528757 PMC4466178

[30] Cohen AS , Rodriguez Z, Warren KK, et al Natural language processing and psychosis: on the need for comprehensive psychometric evaluation. Schizophr Bull. 2022;48(5):939–948. doi:10.1093/schbul/sbac051 35738008 PMC9434462

[31] Hitczenko K , Cowan HR, Goldrick M, Mittal VA. Racial and ethnic biases in computational approaches to psychopathology. Schizophr Bull. 2022;48(2):285–288. doi:10.1093/schbul/sbab131 34729605 PMC8886581

[32] Cohen AS , Elvevåg B. Automated computerized analysis of speech in psychiatric disorders. Curr Opin Psychiatry. 2014;27(3):203–209. doi:10.1097/YCO.0000000000000056 24613984 PMC4212642

[33] Corcoran CM , Mittal VA, Bearden CE, et al Language as a biomarker for psychosis: a natural language processing approach. Schizophr Res. 2020;226:158–166. doi:10.1016/j.schres.2020.04.032 32499162 PMC7704556

[34] Hitczenko K , Mittal VA, Goldrick M. Understanding language abnormalities and associated clinical markers in psychosis: the promise of computational methods. Schizophr Bull. 2021;47(2):344–362. doi:10.1093/schbul/sbaa141 33205155 PMC8480175

[35] Cohen AS , Cox CR, Le TP, et al Using machine learning of computerized vocal expression to measure blunted vocal affect and alogia. NPJ Schizophr. 2020;6(1):26. doi:10.1038/s41537-020-00115-2 32978400 PMC7519104

[36] Fulford D , Mote J, Gonzalez R, et al Smartphone sensing of social interactions in people with and without schizophrenia. J Psychiatr Res. 2021;137:613–620. doi:10.1016/j.jpsychires.2020.11.002 33190842 PMC8084875

[37] Irving J , Patel R, Oliver D, et al Using natural language processing on electronic health records to enhance detection and prediction of psychosis risk. Schizophr Bull. 2021;47(2):405–414. doi:10.1093/schbul/sbaa126 33025017 PMC7965059

[38] Diaz-Asper C , Hauglid MK, Chandler C, Cohen AS, Foltz PW, Elvevåg B. A framework for language technologies in behavioral research and clinical applications: ethical challenges, implications, and solutions. Am Psychol. 2024;79(1):79–91. doi:10.1037/amp0001195 38236217

[39] Kay SR , Fiszbein A, Opler LA. The Positive and Negative Syndrome Scale (PANSS) for schizophrenia. Schizophr Bull. 1987;13(2):261–276. doi:10.1093/schbul/13.2.261 3616518

[40] Rekhi G , Alphs L, Ang MS, Lee J. Clinical utility of the Negative Symptom Assessment-16 in individuals with schizophrenia. Eur Neuropsychopharmacol. 2019;29(12):1433–1441. doi:10.1016/j.euroneuro.2019.10.009 31761524

[41] Green MF , Kern RS, Braff DL, Mintz J. Neurocognitive deficits and functional outcome in schizophrenia: are we measuring the “right stuff?”. Schizophr Bull. 2000;26(1):119–136. doi:10.1093/oxfordjournals.schbul.a033430 10755673

[42] Primack BA , Shensa A, Sidani JE, et al Social media use and perceived social isolation among young adults in the U.S. Am J Prev Med. 2017;53(1):1–8. doi:10.1016/j.amepre.2017.01.010 28279545 PMC5722463

[43] Paquin V , Ackerman RA, Depp CA, Moore RC, Harvey PD, Pinkham AE. Media use and its associations with paranoia in schizophrenia and bipolar disorder: ecological momentary assessment. *JMIR Ment Health*. 2024;11:1–14. doi:10.2196/59198 PMC11238023 38967418

[44] Parrish EM , Depp CA, Moore RC, et al Emotional determinants of life-space through GPS and ecological momentary assessment in schizophrenia: what gets people out of the house? Schizophr Res. 2020;224:67–73. doi:10.1016/j.schres.2020.10.002 33289659 PMC13085614

[45] Harvey PD , Miller ML, Moore RC, Depp CA, Parrish EM, Pinkham AE. Capturing clinical symptoms with ecological momentary assessment: convergence of momentary reports of psychotic and mood symptoms with diagnoses and standard clinical assessments. Innov Clin Neurosci. 2021;18(1-3):24–30. PMC8195558 34150360

[46] Browne J , Harvey P, Buchanan R, et al A longitudinal examination of real-world sedentary behavior in adults with schizophrenia-spectrum disorders in a clinical trial of combined oxytocin and cognitive behavioral social skills training. Behav Sci. 2022;12(3):60. doi:10.3390/bs12030060 35323379 PMC8945120

[47] Parrish EM , Harvey PD, Ackerman RA, et al Time-course and convergence of positive and negative moods in participants with schizophrenia: an ecological momentary assessment study. J Psychiatr Res. 2023;159:76–81. doi:10.1016/j.jpsychires.2023.01.026 36689853

[48] Parrish EM , Chalker S, Cano M, et al Ecological momentary assessment of social approach and avoidance motivations in serious mental illness: connections to suicidal ideation and symptoms. Arch Suicide Res. 2024;28(1):123–140. doi:10.1080/13811118.2022.2137445 36377277 PMC10183051

[49] Depp CA , Moore RC, Perivoliotis D, Holden JL, Swendsen J, Granholm EL. Social behavior, interaction appraisals, and suicidal ideation in schizophrenia: the dangers of being alone. Schizophr Res. 2016;172(1-3):195–200. doi:10.1016/j.schres.2016.02.028 26948502 PMC4915940

[50] Ward T , Rus-Calafell M, Ramadhan Z, et al AVATAR therapy for distressing voices: a comprehensive account of therapeutic targets. Schizophr Bull. 2020;46(5):1038–1044. doi:10.1093/schbul/sbaa061 32372082 PMC7505185

[51] Li W , Zlatanova S. Significant geo-social group discovery over location-based social network. Sensors. 2021;21(13):4551. doi:10.3390/s21134551 34283106 PMC8271907

[52] Raugh IM , James SH, Gonzalez CM, et al Geolocation as a digital phenotyping measure of negative symptoms and functional outcome. Schizophr Bull. 2020;46(6):1596–1607. doi:10.1093/schbul/sbaa121 32851401 PMC7751192

[53] Strauss GP , Bartolomeo LA, Harvey PD. Avolition as the core negative symptom in schizophrenia: relevance to pharmacological treatment development. NPJ Schizophr. 2021;7(1):16. doi:10.1038/s41537-021-00145-4 33637748 PMC7910596

[54] Strassnig MT , Miller ML, Moore R, Depp CA, Pinkham AE, Harvey PD. Evidence for avolition in bipolar disorder? A 30-day ecological momentary assessment comparison of daily activities in bipolar disorder and schizophrenia. Psychiatry Res. 2021;300:113924. doi:10.1016/j.psychres.2021.113924 33848963 PMC8141033

[55] McCreadie RG , Kelly C, Connolly M, et al Dietary improvement in people with schizophrenia: randomised controlled trial. Br J Psychiatry. 2005;187(4):346–351. doi:10.1192/bjp.187.4.346 16199794

[56] Strassnig MT , Harvey PD, Miller ML, Depp CA, Granholm E. Real world sedentary behavior and activity levels in patients with schizophrenia and controls: an ecological momentary assessment study. Ment Health Phys Act. 2021;20:100364. doi:10.1016/j.mhpa.2020.100364 PMC8247127 34221125

[57] Strassnig M , Kotov R, Fochtmann L, Kalin M, Bromet EJ, Harvey PD. Associations of independent living and labor force participation with impairment indicators in schizophrenia and bipolar disorder at 20-year follow-up. Schizophr Res. 2018;197:150–155. doi:10.1016/j.schres.2018.02.009 29472164 PMC6098976

[58] Axelrod BN , Goldman RS, Alphs LD. Validation of the 16-item negative symptom assessment. J Psychiatr Res. 1993;27(3):253–258. doi:10.1016/0022-3956(93)90036-2 7905033

[59] Luck SJ , Hahn B, Leonard CJ, Gold JM. The hyperfocusing hypothesis: a new account of cognitive dysfunction in schizophrenia. Schizophr Bull. 2019;45(5):991–1000. doi:10.1093/schbul/sbz063 31317191 PMC6737469

[60] Gould F , Sabbag S, Durand D, Patterson TL, Harvey PD. Self-assessment of functional ability in schizophrenia: milestone achievement and its relationship to accuracy of self-evaluation. Psychiatry Res. 2013;207(1-2):19–24. doi:10.1016/j.psychres.2013.02.035 23537844 PMC3640769

[61] Durand D , Strassnig MT, Moore RC, et al Self-reported social functioning and social cognition in schizophrenia and bipolar disorder: using ecological momentary assessment to identify the origin of bias. Schizophr Res. 2021;230:17–23. doi:10.1016/j.schres.2021.02.011 33667854 PMC8222067

[62] Harvey PD , Howanitz E, Parrella M, et al Symptoms, cognitive functioning, and adaptive skills in geriatric patients with lifelong schizophrenia: a comparison across treatment sites. Am J Psychiatry. 1998;155(8):1080–1086. doi:10.1176/ajp.155.8.1080 9699697

[63] Sabbag S , Twamley EM, Vella L, Heaton RK, Patterson TL, Harvey PD. Assessing everyday functioning in schizophrenia: not all informants seem equally informative. Schizophr Res. 2011;131(1–3):250–255. doi:10.1016/j.schres.2011.05.003 21620682 PMC3159808

[64] Harvey CD , Moore R C, Depp CA, Ackerman RA, Pinkham AE, Harvey PD. The association of momentary sad moods, concurrent productive behaviour, and global functional outcomes: a 30-day ecological momentary assessment study of people with bipolar illness. Cogn Neuropsychiatry. 2022;27(5):342–355. doi:10.1080/13546805.2022.2070464 35499098

[65] Dalkner N , Moore RC, Depp CA, Ackerman RA, Pinkham AE, Harvey PD. Negative mood states as a correlate of cognitive performance and self-assessment of cognitive performance in bipolar disorder versus schizophrenia. Schizophr Res. 2023;252:1–9. doi:10.1016/j.schres.2022.12.034 36608492 PMC9974828

[66] Jones SE , Moore RC, Depp CA, Ackerman RA, Pinkham AE, Harvey PD. Daily Ecological momentary assessments of happy and sad moods in people with schizophrenia and bipolar disorders: what do participants who are never sad think about their activities and abilities? Schizophr Res Cogn. 2021;26:100202. doi:10.1016/j.scog.2021.100202 34189061 PMC8219985

[67] Strauss GP , Raugh IM, Zhang L, et al Validation of accelerometry as a digital phenotyping measure of negative symptoms in schizophrenia. Schizophrenia. 2022;8(1):37. doi:10.1038/s41537-022-00241-z 35853890 PMC9261099

[68] Martinuzzi LJ , Strassnig MT, Depp CA, et al A closer look at avolition in schizophrenia and bipolar disorder: persistence of different types of activities over time. Schizophr Res. 2022;250:188–195. doi:10.1016/j.schres.2022.11.019 36436498 PMC9810384

[69] Heron KE , Everhart RS, McHale SM, Smyth JM. Using Mobile-Technology-Based Ecological Momentary Assessment (EMA) methods with youth: a systematic review and recommendations. J Pediatr Psychol. 2017;42(10):1087–1107. doi:10.1093/jpepsy/jsx078 28475765

[70] Jones SE , Moore RC, Pinkham AE, Depp CA, Granholm E, Harvey PD. A cross-diagnostic study of adherence to ecological momentary assessment: comparisons across study length and daily survey frequency find that early adherence is a potent predictor of study-long adherence. Pers Med Psychiatry. 2021;29-30:100085. doi:10.1016/j.pmip.2021.100085 34541425 PMC8442600

[71] Depp CA , Kim DH, Vergel De Dios L, Wang V, Ceglowski J. A pilot study of mood ratings captured by mobile phone versus paper-and-pencil mood charts in bipolar disorder. J Dual Diagn. 2012;8(4):326–332. doi:10.1080/15504263.2012.723318 23646035 PMC3640573

[72] Raugh IM , James SH, Gonzalez CM, et al Digital phenotyping adherence, feasibility, and tolerability in outpatients with schizophrenia. J Psychiatr Res. 2021;138:436–443. doi:10.1016/j.jpsychires.2021.04.022 33964681 PMC8192468

[73] Moran EK , Culbreth AJ, Barch DM. Anhedonia in schizophrenia. In: Pizzagalli DA, ed. Anhedonia: Preclinical, Translational, and Clinical Integration. Vol 58. Current Topics in Behavioral Neurosciences. Springer International Publishing; 2022:129–145. doi:10.1007/7854_2022_321 35503596

[74] Gard DE , Sanchez AH, Cooper K, Fisher M, Garrett C, Vinogradov S. Do people with schizophrenia have difficulty anticipating pleasure, engaging in effortful behavior, or both? J Abnorm Psychol. 2014;123(4):771–782. doi:10.1037/abn0000005 25133986 PMC4227944

[75] Kumari S , Mph M, Malik M, Florival MDC, Manalai MDP. An assessment of five (PANSS, SAPS, SANS, NSA-16, CGI-SCH) commonly used symptoms rating scales in schizophrenia and comparison to newer scales (CAINS, BNSS). J Addict Res Ther. 2017;08(03):1–7. doi:10.4172/2155-6105.1000324 PMC5805140 29430333

[76] Mintzer J , Lanctôt KL, Scherer RW, et al; ADMET 2 Research Group. Effect of methylphenidate on apathy in patients with Alzheimer disease: the ADMET 2 randomized clinical trial. JAMA Neurol. 2021;78(11):1324–1332. doi:10.1001/jamaneurol.2021.3356 34570180 PMC8477302

[77] Kring AM , Barch DM. The motivation and pleasure dimension of negative symptoms: neural substrates and behavioral outputs. Eur Neuropsychopharmacol. 2014;24(5):725–736. doi:10.1016/j.euroneuro.2013.06.007 24461724 PMC4020953

[78] Green MF , Horan WP. Effort-based decision making in schizophrenia: evaluation of paradigms to measure motivational deficits: table 1. Schizophr Bull. 2015;41(5):1021–1023. doi:10.1093/schbul/sbv084 26108868 PMC4535648

[79] Krystal AD , Pizzagalli DA, Smoski M, et al A randomized proof-of-mechanism trial applying the ‘fast-fail’ approach to evaluating κ-opioid antagonism as a treatment for anhedonia. Nat Med. 2020;26(5):760–768. doi:10.1038/s41591-020-0806-7 32231295 PMC9949770

[80] Rizvi SJ , Quilty LC, Sproule BA, Cyriac A, Michael Bagby R, Kennedy SH. Development and validation of the Dimensional Anhedonia Rating Scale (DARS) in a community sample and individuals with major depression. Psychiatry Res. 2015;229(1–2):109–119. doi:10.1016/j.psychres.2015.07.062 26250147

[81] Gard DE , Kring AM, Gard MG, Horan WP, Green MF. Anhedonia in schizophrenia: distinctions between anticipatory and consummatory pleasure. Schizophr Res. 2007;93(1-3):253–260. doi:10.1016/j.schres.2007.03.008 17490858 PMC1986826

[82] Edwards CJ , Cella M, Emsley R, Tarrier N, Wykes THM. Exploring the relationship between the anticipation and experience of pleasure in people with schizophrenia: an experience sampling study. Schizophr Res. 2018;202:72–79. doi:10.1016/j.schres.2018.06.040 30007868 PMC6294730

[83] Shovestul B , Saxena A, Reda S, et al Social affective forecasting and social anhedonia in schizophrenia-spectrum disorders: a daily diary study. Schizophrenia. 2022;8(1):97. doi:10.1038/s41537-022-00310-3 36376338 PMC9663197

[84] Brenner CJ , Ben-Zeev D. Affective forecasting in schizophrenia: comparing predictions to real-time Ecological Momentary Assessment (EMA) ratings. Psychiatr Rehabil J. 2014;37(4):316–320. doi:10.1037/prj0000105 25496200

[85] Moran EK , Culbreth AJ, Barch DM. Ecological momentary assessment of negative symptoms in schizophrenia: relationships to effort-based decision making and reinforcement learning. J Abnorm Psychol. 2017;126(1):96–105. doi:10.1037/abn0000240 27893230 PMC5433621

[86] Culbreth AJ , Moran EK, Kandala S, Westbrook A, Barch DM. Effort, avolition, and motivational experience in schizophrenia: analysis of behavioral and neuroimaging data with relationships to daily motivational experience. Clin Psychol Sci. 2020;8(3):555–568. doi:10.1177/2167702620901558 33758684 PMC7983405

[87] Sanchez AH , Lavaysse LM, Starr JN, Gard DE. Daily life evidence of environment-incongruent emotion in schizophrenia. Psychiatry Res. 2014;220(1-2):89–95. doi:10.1016/j.psychres.2014.07.041 25124684 PMC4252781

[88] Granholm E , Holden JL, Mikhael T, et al What do people with schizophrenia do all day? Ecological momentary assessment of real-world functioning in schizophrenia. Schizophr Bull. 2019;46:sbz070. doi:10.1093/schbul/sbz070 PMC7442321 31504955

[89] Merchant JT , Moran EK, Strube MJ, Barch DM. Correlates of real-world goal-directed behavior in schizophrenia. Psychol Med. 2023;53(6):2409–2417. doi:10.1017/S0033291721004281 34763732 PMC11578271

[90] Nuechterlein KH , Green MF, Kern RS, et al The MATRICS consensus cognitive battery, part 1: test selection, reliability, and validity. Am J Psychiatry. 2008;165(2):203–213. doi:10.1176/appi.ajp.2007.07010042 18172019

